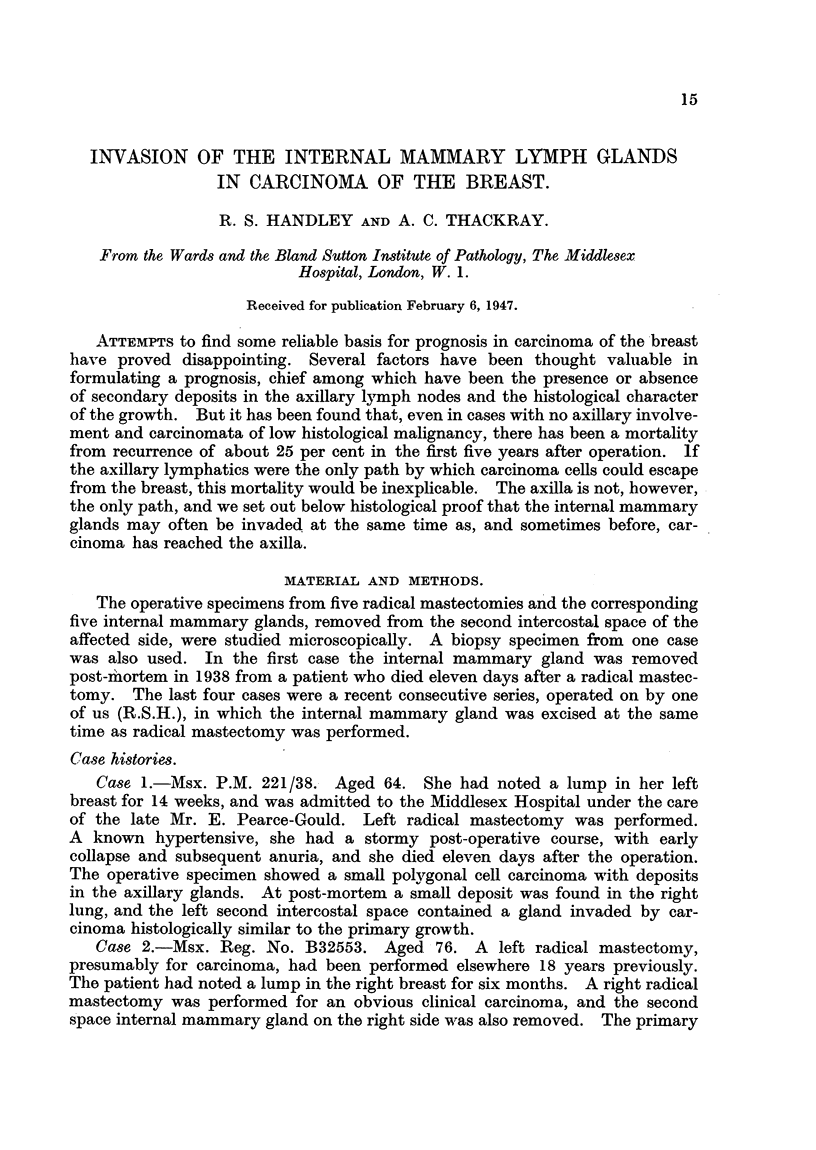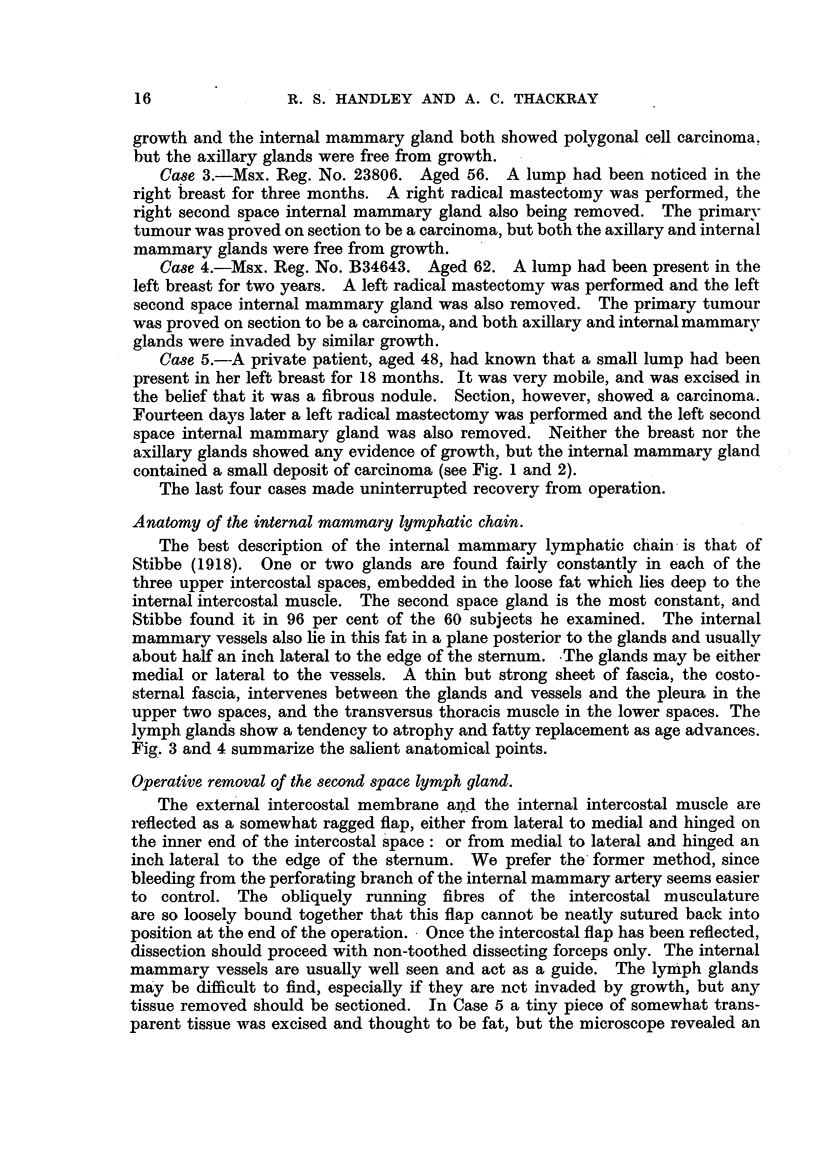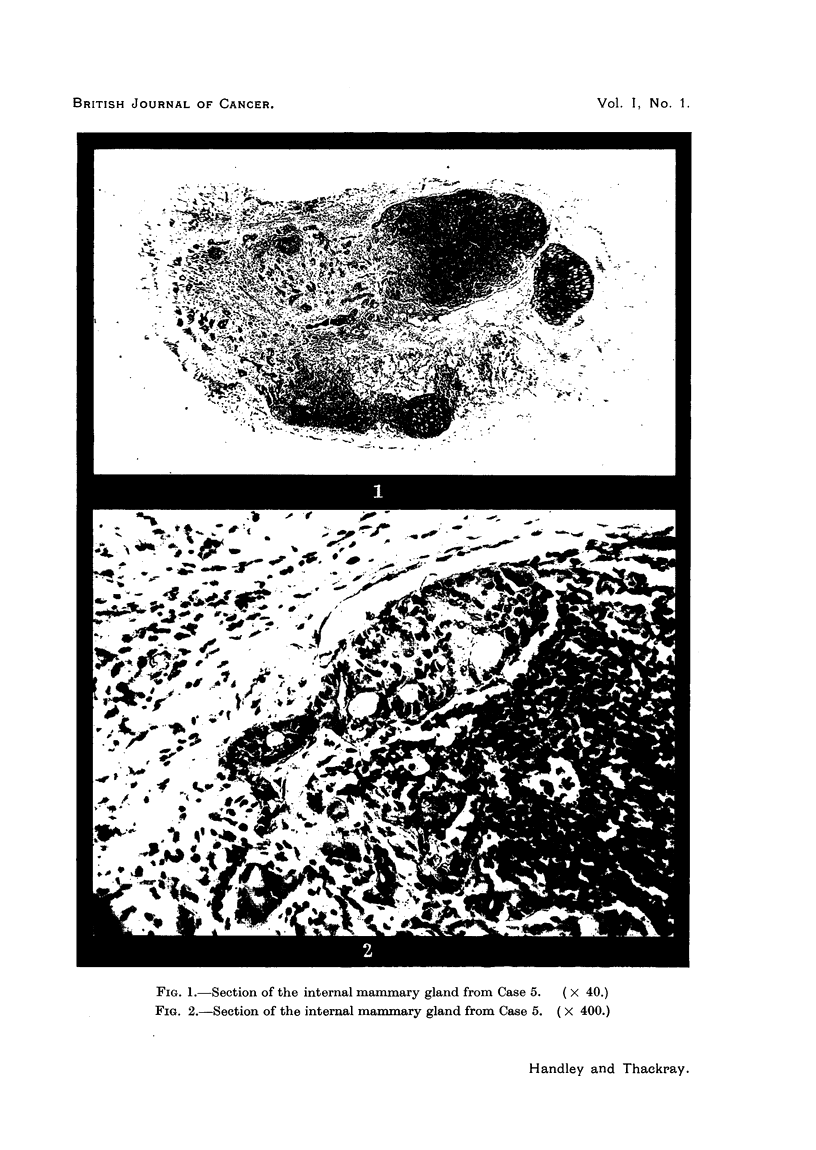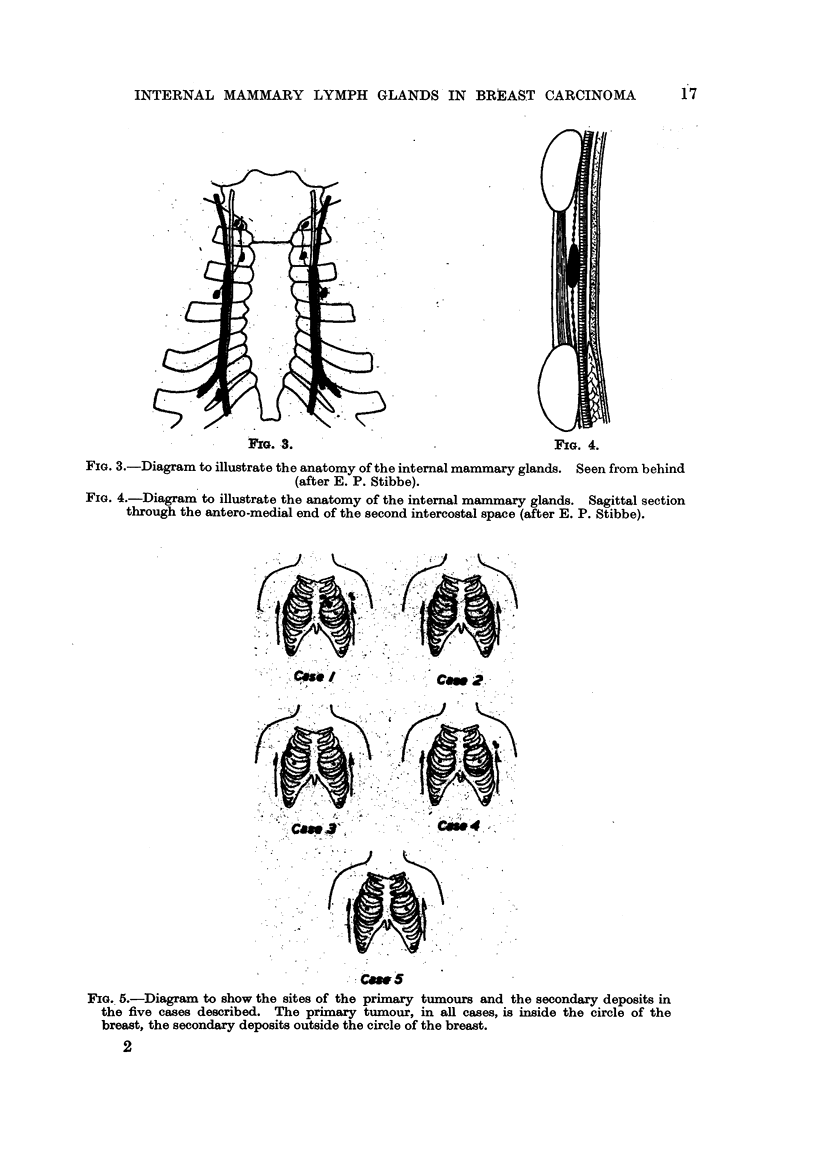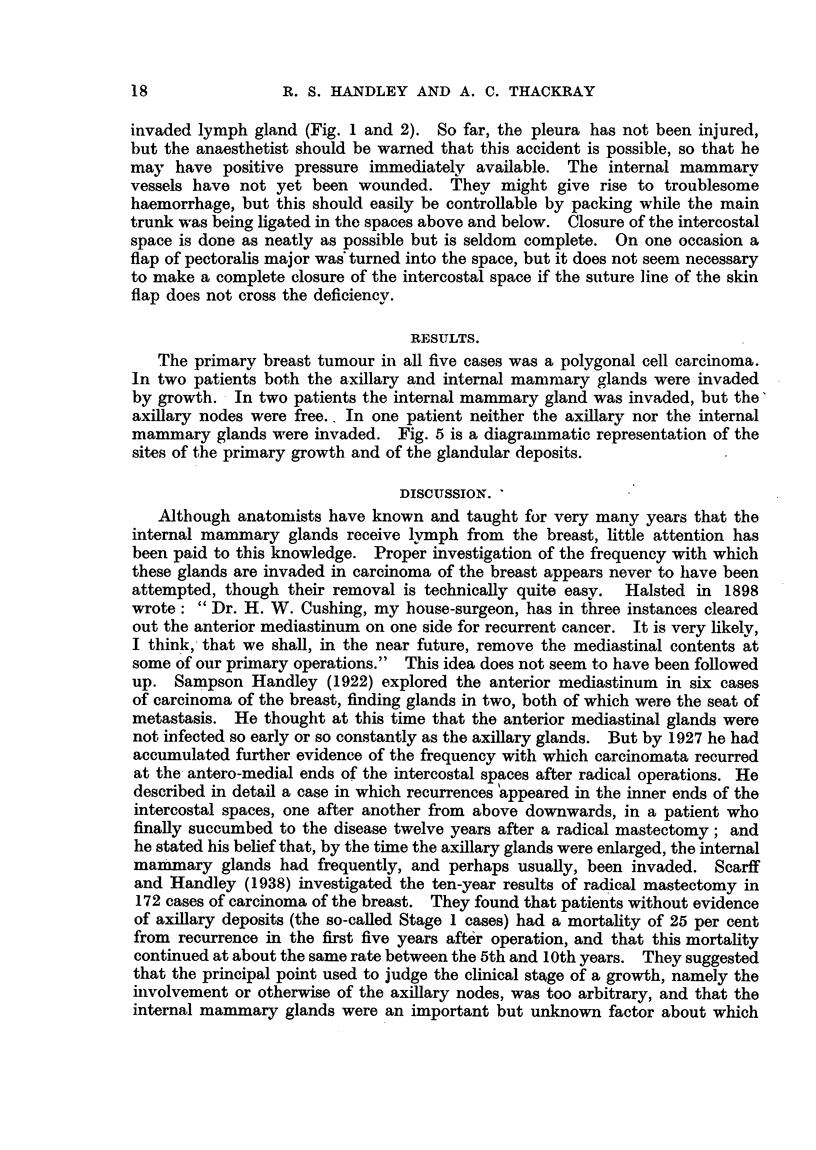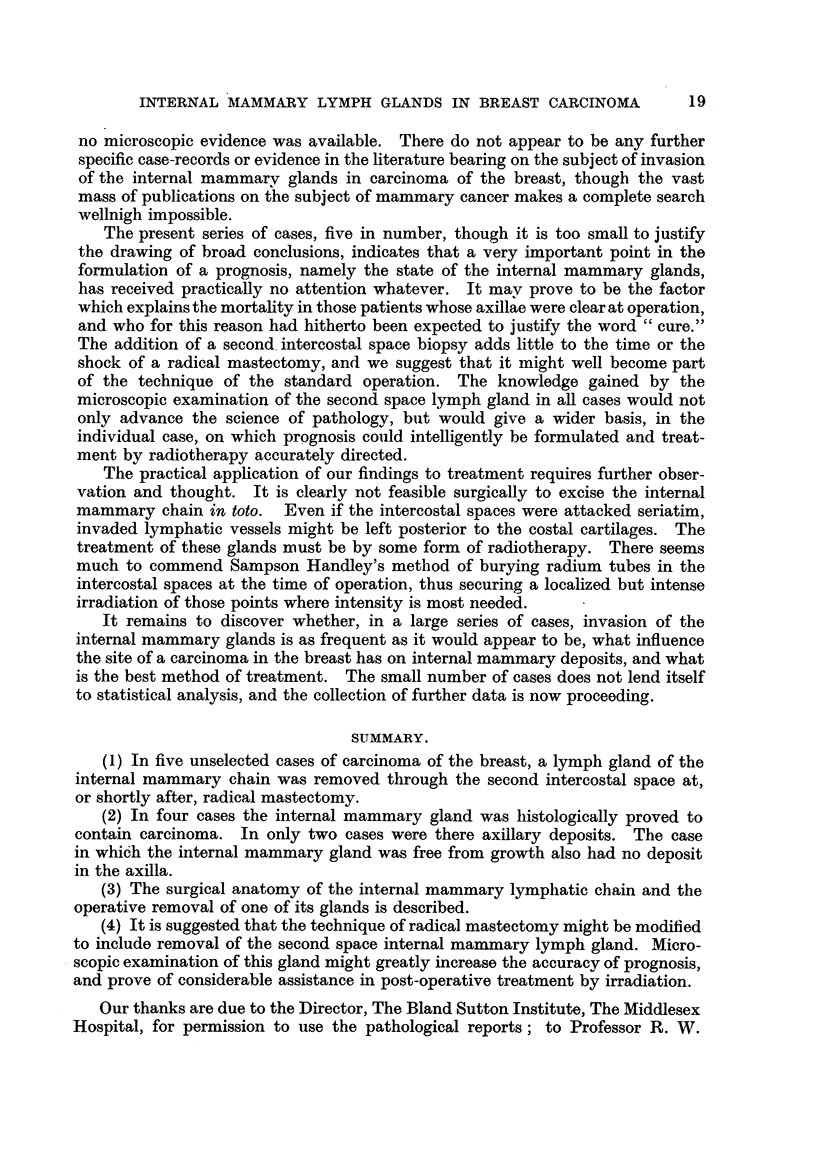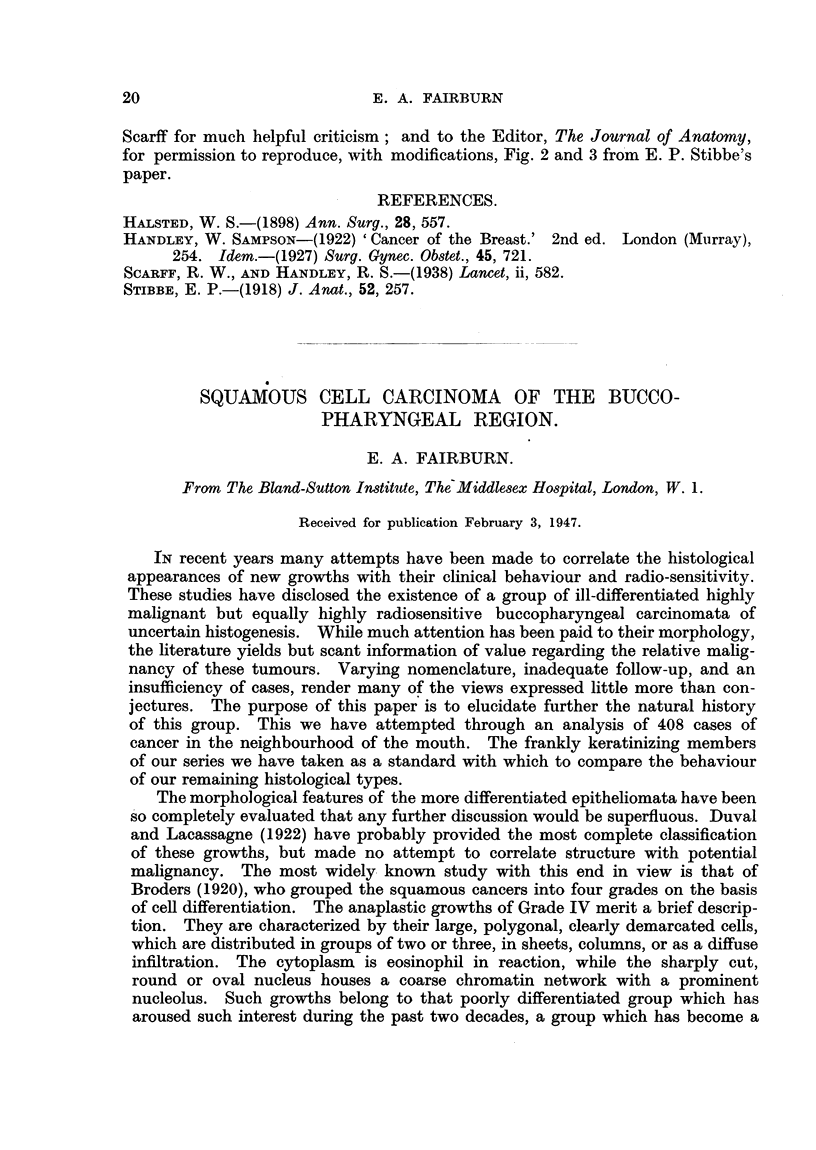# Invasion of the Internal Mammary Lymph Glands in Carcinoma of the Breast

**DOI:** 10.1038/bjc.1947.2

**Published:** 1947-03

**Authors:** R. S. Handley, A. C. Thackray

## Abstract

**Images:**


### INVASION OF THE INTERNAL MAMMARY LYMPH GLANDS IN CARCINOMA OF THE BREAST.

R. S. HANDLEY AND A. C. THACKRAY.

*From the Wards and the Bland Sutton Institute of Pathology, The Middlesex Hospital, London, W. 1.*

Received for publication February 6, 1947.

ATTEMPTS to find some reliable basis for prognosis in carcinoma of the breast have proved disappointing. Several factors have been thought valuable in formulating a prognosis, chief among which have been the presence or absence of secondary deposits in the axillary lymph nodes and the histological character of the growth. But it has been found that, even in cases with no axillary involvement and carcinomata of low histological malignancy, there has been a mortality from recurrence of about 25 per cent in the first five years after operation. If the axillary lymphatics were the only path by which carcinoma cells could escape from the breast, this mortality would be inexplicable. The axilla is not, however, the only path, and we set out below histological proof that the internal mammary glands may often be invaded at the same time as, and sometimes before, carcinoma has reached the axilla.

### MATERIAL AND METHODS.

The operative specimens from five radical mastectomies and the corresponding five internal mammary glands, removed from the second intercostal space of the affected side, were studied microscopically. A biopsy specimen from one case was also used. In the first case the internal mammary gland was removed post-mortem in 1938 from a patient who died eleven days after a radical mastectomy. The last four cases were a recent consecutive series, operated on by one of us (R.S.H.), in which the internal mammary gland was excised at the same time as radical mastectomy was performed.

### *Case histories.*

*Case* 1.—Msx. P.M. 221/38. Aged 64. She had noted a lump in her left breast for 14 weeks, and was admitted to the Middlesex Hospital under the care of the late Mr. E. Pearce-Gould. Left radical mastectomy was performed. A known hypertensive, she had a stormy post-operative course, with early collapse and subsequent anuria, and she died eleven days after the operation. The operative specimen showed a small polygonal cell carcinoma with deposits in the axillary glands. At post-mortem a small deposit was found in the right lung, and the left second intercostal space contained a gland invaded by carcinoma histologically similar to the primary growth.

*Case* 2.—Msx. Reg. No. B32553. Aged 76. A left radical mastectomy, presumably for carcinoma, had been performed elsewhere 18 years previously. The patient had noted a lump in the right breast for six months. A right radical mastectomy was performed for an obvious clinical carcinoma, and the second space internal mammary gland on the right side was also removed. The primary growth and the internal mammary gland both showed polygonal cell carcinoma, but the axillary glands were free from growth.

*Case* 3.—Msx. Reg. No. 23806. Aged 56. A lump had been noticed in the right breast for three months. A right radical mastectomy was performed, the right second space internal mammary gland also being removed. The primary tumour was proved on section to be a carcinoma, but both the axillary and internal mammary glands were free from growth.

*Case* 4.—Msx. Reg. No. B34643. Aged 62. A lump had been present in the left breast for two years. A left radical mastectomy was performed and the left second space internal mammary gland was also removed. The primary tumour was proved on section to be a carcinoma, and both axillary and internal mammary glands were invaded by similar growth.

*Case* 5.—A private patient, aged 48, had known that a small lump had been present in her left breast for 18 months. It was very mobile, and was excised in the belief that it was a fibrous nodule. Section, however, showed a carcinoma. Fourteen days later a left radical mastectomy was performed and the left second space internal mammary gland was also removed. Neither the breast nor the axillary glands showed any evidence of growth, but the internal mammary gland contained a small deposit of carcinoma (see Fig. 1 and 2).

The last four cases made uninterrupted recovery from operation.

*Anatomy of the internal mammary lymphatic chain.*

The best description of the internal mammary lymphatic chain is that of Stibbe (1918). One or two glands are found fairly constantly in each of the three upper intercostal spaces, embedded in the loose fat which lies deep to the internal intercostal muscle. The second space gland is the most constant, and Stibbe found it in 96 per cent of the 60 subjects he examined. The internal mammary vessels also lie in this fat in a plane posterior to the glands and usually about half an inch lateral to the edge of the sternum. The glands may be either medial or lateral to the vessels. A thin but strong sheet of fascia, the costo-sternal fascia, intervenes between the glands and vessels and the pleura in the upper two spaces, and the transversus thoracis muscle in the lower spaces. The lymph glands show a tendency to atrophy and fatty replacement as age advances. Fig. 3 and 4 summarize the salient anatomical points.

*Operative removal of the second space lymph gland.*

The external intercostal membrane and the internal intercostal muscle are reflected as a somewhat ragged flap, either from lateral to medial and hinged on the inner end of the intercostal space : or from medial to lateral and hinged an inch lateral to the edge of the sternum. We prefer the former method, since bleeding from the perforating branch of the internal mammary artery seems easier to control. The obliquely running fibres of the intercostal musculature are so loosely bound together that this flap cannot be neatly sutured back into position at the end of the operation. Once the intercostal flap has been reflected, dissection should proceed with non-toothed dissecting forceps only. The internal mammary vessels are usually well seen and act as a guide. The lymph glands may be difficult to find, especially if they are not invaded by growth, but any tissue removed should be sectioned. In Case 5 a tiny piece of somewhat trans-parent tissue was excised and thought to be fat, but the microscope revealed an

1

2

FIG. 1.—Section of the internal mammary gland from Case 5. (× 40.)

FIG. 2.—Section of the internal mammary gland from Case 5. (× 400.)

FIG. 3.

FIG. 4.

FIG. 3.—Diagram to illustrate the anatomy of the internal mammary glands. Seen from behind (after E. P. Stibbe).

FIG. 4.—Diagram to illustrate the anatomy of the internal mammary glands. Sagittal section through the antero-medial end of the second intercostal space (after E. P. Stibbe).

FIG. 5.—Diagram to show the sites of the primary tumours and the secondary deposits in the five cases described. The primary tumour, in all cases, is inside the circle of the breast, the secondary deposits outside the circle of the breast.

invaded lymph gland (Fig. 1 and 2). So far, the pleura has not been injured, but the anaesthetist should be warned that this accident is possible, so that he may have positive pressure immediately available. The internal mammary vessels have not yet been wounded. They might give rise to troublesome haemorrhage, but this should easily be controllable by packing while the main trunk was being ligated in the spaces above and below. Closure of the intercostal space is done as neatly as possible but is seldom complete. On one occasion a flap of pectoralis major was turned into the space, but it does not seem necessary to make a complete closure of the intercostal space if the suture line of the skin flap does not cross the deficiency.

### RESULTS.

The primary breast tumour in all five cases was a polygonal cell carcinoma. In two patients both the axillary and internal mammary glands were invaded by growth. In two patients the internal mammary gland was invaded, but the axillary nodes were free. In one patient neither the axillary nor the internal mammary glands were invaded. Fig. 5 is a diagrammatic representation of the sites of the primary growth and of the glandular deposits.

### DISCUSSION.

Although anatomists have known and taught for very many years that the internal mammary glands receive lymph from the breast, little attention has been paid to this knowledge. Proper investigation of the frequency with which these glands are invaded in carcinoma of the breast appears never to have been attempted, though their removal is technically quite easy. Halsted in 1898 wrote: " Dr. H. W. Cushing, my house-surgeon, has in three instances cleared out the anterior mediastinum on one side for recurrent cancer. It is very likely, I think, that we shall, in the near future, remove the mediastinal contents at some of our primary operations." This idea does not seem to have been followed up. Sampson Handley (1922) explored the anterior mediastinum in six cases of carcinoma of the breast, finding glands in two, both of which were the seat of metastasis. He thought at this time that the anterior mediastinal glands were not infected so early or so constantly as the axillary glands. But by 1927 he had accumulated further evidence of the frequency with which carcinomata recurred at the antero-medial ends of the intercostal spaces after radical operations. He described in detail a case in which recurrences appeared in the inner ends of the intercostal spaces, one after another from above downwards, in a patient who finally succumbed to the disease twelve years after a radical mastectomy; and he stated his belief that, by the time the axillary glands were enlarged, the internal mammary glands had frequently, and perhaps usually, been invaded. Scarff and Handley (1938) investigated the ten-year results of radical mastectomy in 172 cases of carcinoma of the breast. They found that patients without evidence of axillary deposits (the so-called Stage 1 cases) had a mortality of 25 per cent from recurrence in the first five years after operation, and that this mortality continued at about the same rate between the 5th and 10th years. They suggested that the principal point used to judge the clinical stage of a growth, namely the involvement or otherwise of the axillary nodes, was too arbitrary, and that the internal mammary glands were an important but unknown factor about which no microscopic evidence was available. There do not appear to be any further specific case-records or evidence in the literature bearing on the subject of invasion of the internal mammary glands in carcinoma of the breast, though the vast mass of publications on the subject of mammary cancer makes a complete search wellnigh impossible.

The present series of cases, five in number, though it is too small to justify the drawing of broad conclusions, indicates that a very important point in the formulation of a prognosis, namely the state of the internal mammary glands, has received practically no attention whatever. It may prove to be the factor which explains the mortality in those patients whose axillae were clear at operation, and who for this reason had hitherto been expected to justify the word " cure." The addition of a second intercostal space biopsy adds little to the time or the shock of a radical mastectomy, and we suggest that it might well become part of the technique of the standard operation. The knowledge gained by the microscopic examination of the second space lymph gland in all cases would not only advance the science of pathology, but would give a wider basis, in the individual case, on which prognosis could intelligently be formulated and treatment by radiotherapy accurately directed.

The practical application of our findings to treatment requires further observation and thought. It is clearly not feasible surgically to excise the internal mammary chain *in toto*. Even if the intercostal spaces were attacked seriatim, invaded lymphatic vessels might be left posterior to the costal cartilages. The treatment of these glands must be by some form of radiotherapy. There seems much to commend Sampson Handley's method of burying radium tubes in the intercostal spaces at the time of operation, thus securing a localized but intense irradiation of those points where intensity is most needed.

It remains to discover whether, in a large series of cases, invasion of the internal mammary glands is as frequent as it would appear to be, what influence the site of a carcinoma in the breast has on internal mammary deposits, and what is the best method of treatment. The small number of cases does not lend itself to statistical analysis, and the collection of further data is now proceeding.

### SUMMARY.

(1) In five unselected cases of carcinoma of the breast, a lymph gland of the internal mammary chain was removed through the second intercostal space at, or shortly after, radical mastectomy.

(2) In four cases the internal mammary gland was histologically proved to contain carcinoma. In only two cases were there axillary deposits. The case in which the internal mammary gland was free from growth also had no deposit in the axilla.

(3) The surgical anatomy of the internal mammary lymphatic chain and the operative removal of one of its glands is described.

(4) It is suggested that the technique of radical mastectomy might be modified to include removal of the second space internal mammary lymph gland. Microscopic examination of this gland might greatly increase the accuracy of prognosis, and prove of considerable assistance in post-operative treatment by irradiation.

Our thanks are due to the Director, The Bland Sutton Institute, The Middlesex Hospital, for permission to use the pathological reports; to Professor R. W.

Scarff for much helpful criticism ; and to the Editor, *The Journal of Anatomy*, for permission to reproduce, with modifications, Fig. 2 and 3 from E. P. Stibbe's paper.

### REFERENCES.

HALSTED, W. S.—(1898) *Ann. Surg.*, **28**, 557.
HANDLEY, W. SAMPSON—(1922) 'Cancer of the Breast.' 2nd ed. London (Murray), 254. *Idem.*—(1927) *Surg. Gynec. Obstet.*, **45**, 721.
SCARFF, R. W., AND HANDLEY, R. S.—(1938) *Lancet*, ii, 582.
STIBBE, E. P.—(1918) *J. Anat.*, **52**, 257.